# Landscape-scale assessments of stable carbon isotopes in soil under diverse vegetation classes in East Africa: application of near-infrared spectroscopy

**DOI:** 10.1007/s11104-017-3418-3

**Published:** 2017-10-16

**Authors:** Leigh Ann Winowiecki, Tor-Gunnar Vågen, Pascal Boeckx, Jennifer A. J. Dungait

**Affiliations:** 1 World Agroforestry Centre (ICRAF), Nairobi, Kenya; 2 Isotope Bioscience Laboratory – ISOFYS, Ghent University, Coupure Links 653, 9000 Gent, Belgium; 3 Rothamsted Research, Sustainable Soils and Grassland Systems Department, North Wyke, Okehampton, Devon EX20 2SB, UK

**Keywords:** Carbon cycling, Landscape scale assessments, Random forest modelling

## Abstract

**Aims:**

Stable carbon isotopes are important tracers used to understand ecological food web processes and vegetation shifts over time. However, gaps exist in understanding soil and plant processes that influence δ^13^C values, particularly across smallholder farming systems in sub-Saharan Africa. This study aimed to develop predictive models for δ^13^C values in soil using near infrared spectroscopy (NIRS) to increase overall sample size. In addition, this study aimed to assess the δ^13^C values between five vegetation classes.

**Methods:**

The Land Degradation Surveillance Framework (LDSF) was used to collect a stratified random set of soil samples and to classify vegetation. A total of 154 topsoil and 186 subsoil samples were collected and analyzed using NIRS, organic carbon (OC) and stable carbon isotopes.

**Results:**

Forested plots had the most negative average δ^13^C values, −26.1‰; followed by woodland, −21.9‰; cropland, −19.0‰; shrubland, −16.5‰; and grassland, −13.9‰. Prediction models were developed for δ^13^C using partial least squares (PLS) regression and random forest (RF) models. Model performance was acceptable and similar with both models. The root mean square error of prediction (RMSEP) values for the three independent validation runs for δ^13^C using PLS ranged from 1.91 to 2.03 compared to 1.52 to 1.98 using RF.

**Conclusions:**

This model performance indicates that NIR can be used to predict δ^13^C in soil, which will allow for landscape-scale assessments to better understand carbon dynamics.

## Introduction

Aboveground vegetation influences belowground carbon dynamics. Optimizing soil organic carbon (SOC) content is recognized as an essential component of ecosystem functioning (Lal 2010; Palm et al. 2007; Vågen et al. 2012). The United Nations Convention to Combat Desertification (UNCCD) and the United Nations Framework Convention on Climate Change (UNFCCC) both recognize that reduced SOC content is a consequence of, and can lead to further land degradation, and ultimately poor land and agricultural productivity. However, understanding the influence of vegetation classes on SOC is still needed, especially in light of progressive degradation of soil and water resources (Vågen et al. 2013a; Vågen and Gumbritch 2012; Verchot et al. 2005). Although SOC is almost universally proposed as the most important soil quality indicator (Amundson et al. 2015; Gregorich et al. 1994), the complexity and extent of SOC dynamics at the landscape scale is still poorly understood. This includes, but is not limited to, understanding the influence of inherent soil properties (e.g. geochemistry, aggregation, texture, etc.) on SOC content as well as the effects of aboveground vegetation types, land management and climate. Furthermore, the impacts of land-use change on SOC dynamics in sub-Saharan African (SSA) ecosystems are still understudied, especially across diverse landscapes, but essential if food production is to keep pace with predicted population growth in the region (Rosegrant and Cline 2003). Assessing the impact of vegetation shifts on SOC dynamics and quantifying SOC turnover rates can improve our understanding of the effects of land-use change from native vegetation to agricultural food production (Schlesinger 1997), as well as the impacts of management shifts introduced by climate smart agriculture (Lipper 2014; Rwehumbiza 2014) and sustainable agricultural intensification (Vanlauwe et al. 2014) on soil health in smallholder farming systems.

## Stable carbon isotopes in soil

Stable carbon isotopes are important tracers used to understand ecological food web processes and vegetation shifts over time. This is because, the majority of plants (trees and broad-leaved crops) use the C3 photosynthetic pathway and have δ^13^C values between –22 and –30‰, while about 15% of plants use the C4 photosynthetic pathway and have less negative δ^13^C values, generally ranging from –10 to – 14‰ (Farquhar 1989); Loomis and Connor 1992). The latter includes the majority of tropical herbs and grasses, including maize which is the major crop grown in many of our study areas.

Understanding differences in photosynthetic pathways is important in the assessment of SOC dynamics, including SOM turnover rates and carbon cycling (Accoe et al. 2002; Bernoux et al. 1998; Ehleringer et al. 2000; Six and Jastrow 2002); to identify vegetative sources of organic matter in the soil (Boutton et al. 1998; Von Fischer and Tieszen 1995; Kindscher and Tieszen 1998; Krull et al. 2006; Puttock et al. 2014, 2012; Roscoe et al. 2001); to address the impact of land conversion on soil condition (Awiti et al. 2008; Schulp and Veldkamp 2008; Vågen et al. 2006b) and to improve the overall understanding of ecosystem function (Staddon 2004). In addition to vegetative shifts, there are several other factors that influence δ^13^C values in soil, including (i) microbial decomposition, (ii) the Suess effect (Balesdent and Mariotti 1996; Roscoe et al. 2001), and (iii) inherent factors such as soil texture and geochemistry (e.g., quantity of iron and aluminum oxides) (Krull et al. 2002; Krull and Skjemstad 2003; Powers and Schlesinger 2002). There are still gaps in our knowledge regarding stable carbon isotope signatures in soils under in diverse systems, due in part to the associated costs, infrastructure requirements and sample preparation time required for stable carbon isotope analysis, which inhibit landscape-scale assessments. However, the interest in stable isotopes is increasing, as is its utility across disciplines. For example, the spatial patterns of vegetative signatures in soil have been mapped using stable carbon isotopes (Boeckx et al. 2006; Wynn and Bird 2007), δ^13^C values in the soil profile have been used as a proxy for SOC stability (Oelbermann and Voroney 2006; Salomé et al. 2010), to trace and quantify erosion (Häring et al. 2013), and δ^13^C values in sediments have been used to determine vegetative sources in depositional environments (Puttock et al. 2012). Compound-specific stable carbon isotope values are increasingly applied to understand different soil processes using a range of organic compounds with contrasting chemistries used as biomarkers (Dungait et al. 2008, 2010). The increase in the use of stable carbon isotopes has created a demand for new, more rapid analysis of plants, soils and sediments in order to obtain the larger datasets needed for landscape-scale ecological assessments.

## Use of near-infrared spectroscopy (NIRS) to predict soil properties

Infrared (IR) is now a well established methodology for the prediction of soil properties such as SOC, pH, base cations and texture (Brown 2007; Brown et al. 2006; Genot et al. 2011; Nocita et al. 2014; Stenberg et al. 2010; Vasques et al. 2009; Viscarra Rossel et al. 2006). In addition to being cost effective, IR spectroscopy allows for estimation of several soil characteristics simultaneously, with minimal sample preparation and no use of chemicals (Brown et al. 2006; Vågen et al. 2006a, b; Terhoeven-Urselmans et al. 2010; Genot et al. 2011). The ability to distinguish between different properties of a wide range of materials using NIR has resulted in a growing field of research across several disciplines, including chemometrics, forage science, soil science and plant science. In soil science, the application of NIR has resulted in a significant lowering of costs associated with measurements of soil properties, which has in turn resulted in significant advances when it comes to landscape-scale assessments of soil.

Soil spectroscopy is the “reflectance part of the electromagentic radiation that interacts with the soil matter across the VIS-NIR_SWIR spectral region” (Ben-Dor and Banin 1995). Specifically, spectra in the near infrared (NIR) range (wavelengths 8000–4000 cm^–1^), can be analyzed to characterize the chemical, physical and mineralogical composition of the soil (Ben-Dor and Banin 1995; Stoner and Baumgardner 1982; Viscarra Rossel et al. 2006). NIRS are influenced not only by the chemistry of the soil but also by its physical structure, making individual (well-defined and narrow) absorption bands at specific wavelengths less pronounced. As NIR absorbance features occur due to both overtones and combination bands of fundamental vibrations of OH, CH, NH, CO, CN and NO bonds in the mid infrared region, the absorbance of light is directly related to frequency or wavelength and corresponds to the difference in energy between two vibrational states (quantum numbers) in molecular bonds. These energy levels are also influenced by surrounding molecules and functional groups, for example, but fundamentally various substances and molecules can be identified due to different absorption patterns in the spectra. For example, spectral regions around 7000 cm^–1^, 5200 cm^–1^ and 4460 cm^–1^ are particularly important for the prediction of SOC (Ben-Dor and Banin 1995). Iron oxides are often represented in the adsorption bands at less then 1000 nm, hydroxyl bonds near 1400 and 1900 nm, clay mineral absorb near 2200, and organic matter absorbs at various wavelengths throughout NIR spectrum (Soriano-Disla et al. 2014; Viscarra Rossel et al. 2016). Few studies have reported important spectral regions for the prediction of other soil properties than SOC. The difference between the application of spectroscopy in soil science versus for example in food sciences, is that that soil properties do not exhibit strong peaks at particular wavelengths, calibration models with reference datasets containing wet chemistry analysis is needed to develop robust predictive models.

The World Agroforestry Centre (ICRAF), with headquarters in Nairobi, Kenya has established a soil IR spectral database that currently has soil spectra from soil samples from across a wide range of landscapes that represent agricultural soils, forested landscapes, wetlands, and savannas (Brown 2007; Towett et al. 2015). These landscapes also represent a wide range of climatic conditions, from sub-humid and humid ecosystems to semi-arid and arid ecosystems in the global tropics. This soil IR database combined with associated reference wet chemistry enables the application of data mining and analysis techniques to explore the potential of IR to predict soil properties and even indices of soil condition (Vågen et al. 2006a, b).

Other studies have assessed the potential for NIR to predict stable carbon isotopes in soil, as it is plausible that NIR spectra should be able to detect the differences in the atomic mass of the carbon isotope (Kleinebecker et al. 2009). For example, Fuentes et al. (2009, 2012) explored the possibility to predict δ^13^C values in soil using NIR spectra from soils collected from a well documented experimental station in Mexico (*n* = 100 soil samples). Using modified partial least squares (MPLS) regression, they developed calibration models with an R^2^ of 0.81 (Fuentes et al. 2012, 2009). Fuentes et al. (2009) used Mahalanobis distance to characterize and discard spectra from the population of soil samples, hence attempting to reduce variability in the soil samples used in the study. While the results presented in Fuentes et al. (2009) were promising, and demonstrated the potential for using NIR to predict δ^13^C, the study represented a dataset with very limited variations in δ^13^C and separate models were developed for soils with plant residues (*n* = 50 range – 16.6 to –23.3‰) and without plant residues (n = 50 range – 19.1 to –22.1‰), which limits the application of these models beyond the specific case study they were developed for. Our study builds on this work to explore the potential for NIR to predict δ^13^C values across a varied set of soils that represent a wide range of chemical and physical characteristics, including different vegetation classes. Kleinebecker et al. (2009) used NIR spectra to predict δ^13^C and δ^15^N in plant tissues. Applying partial least squares (PLS) regression they developed calibration models obtaining positive results with R^2^ values of 0.89 and 0.99 for δ^13^C and δ^15^N, respectively (Kleinebecker et al. 2009). While NIR has long been used to determine protein quality in forages (Marten et al. 1983). Clark et al. (1995) assessed the potential to determine carbon isotope composition using NIR in various genotypes and cultivars of forage species in the USA. Their study used PLS regression models for each cultivar and obtained R^2^ values between 0.69 to 0.93 for δ^13^C calibration models, further highlighting the utility of NIR to predict stable carbon isotopic content (Clark et al. 1995) in plant material.

Recent advances in big data analytics and ensemble learning methods allow for the development of predictive models that are stable across a range of soil functional operating ranges. In this paper we present a case study where we use NIR spectroscopy of soils to develop predictive models for SOC and δ^13^C, exploring the application of this approach in the scaling of soil analysis to landscape level assessments of soil health. Specific objectives of this study include: 1) Assess the potential for NIRS to predict δ^13^C using a diverse set of soil samples; 2) Compare different predictive models (e.g., PLS and RF) and 3) Better understand stable carbon isotope variation across various vegetation classes and smallholder farming systems in SSA.

## Materials and methods

### Soil sampling

**Fig. 1:**
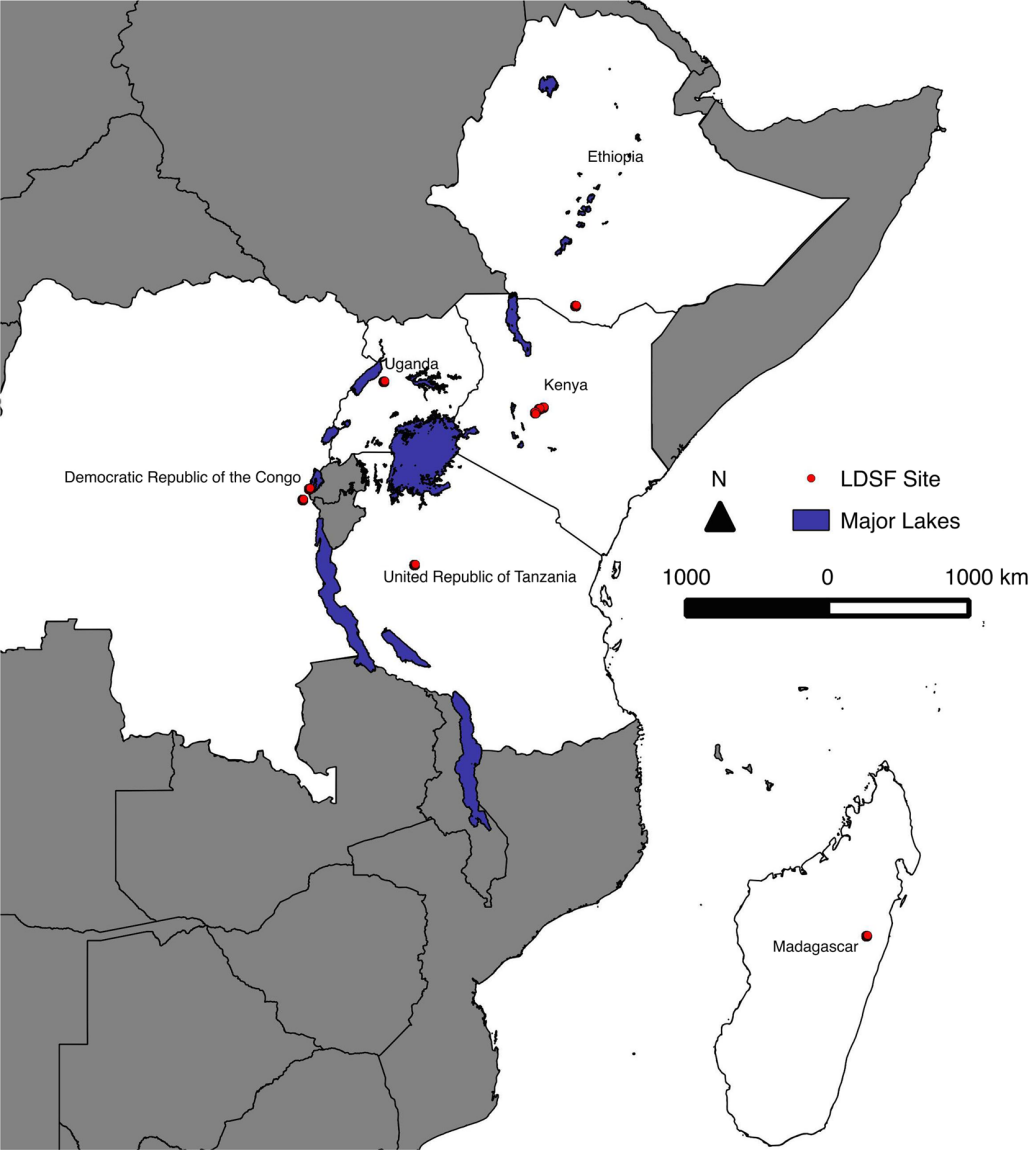
Location of the nine LDSF sites used in the study (red circles)

Biophysical field surveys and soil sampling were conducted in nine-100 km^2^ sites using the Land Degradation Surveillance Framework (LDSF). Top (*n* = 156) and sub (*n* = 184) soil samples from nine LDSF sites across Ethiopia (Mega), Kenya (OlLentille, Mpala, Kipsing), Democratic Republic of Congo (DRC) (Luhihi, Burhale), Uganda (Hoima), Madagascar (Didy) and Tanzania (Mbola) were included in the study (Fig. 1). The LDSF uses a spatially stratified random sampling design (Vågen et al. 2013b) with 160 sampling plots, each 1000 m^2^, across 16 spatially stratified clusters (10 plots in each cluster), with 4 subplots (100 m^2^) within each sampling plot. Measurements and observations were made at the subplot and plot levels, respectively. Land use was classified at each plot using a simplified version of the FAO Land Cover Classification System (LCCS) (Di Gregorio and Jansen 1998), into three distinct vegetation classes: 1) primarily vegetated, forest; (2) primarily vegetated, woodland; (3) primarily vegetated, shrubland; (4) primarily vegetated, grassland; and (5) primarily vegetated, cropland (Di Gregorio and Jansen 1998). Altitude was recorded at the plot level. Tree counts were made at the subplot level and then averaged at the plot level. Soil samples were collected from each of the 160 plots by compositing soil samples from the four subplots within each plot for topsoil (0–20 cm) and subsoil (20–50 cm). For most of the sites, we analyzed soil samples from one reference plot from each cluster, for a total of 16 topsoil samples and 16 subsoil samples (unless there were depth restrictions), providing a total of 30 soil samples from Luhihi, Burhale, and OlLentille, 31 soil samples from Mpala, 32 soil samples from Kipsing, Mega and Hoima. However, two reference plots per cluster were used for Didy and Mbola, providing 64 and 59 soil samples from these sites, respectively.

### Laboratory analysis

Soil samples were air-dried and sieved to 2 mm. Airdried soil samples were scanned in duplicate in nearinfrared spectral range (wavelengths between 8000 to 4000 cm^–1^) with a resolution of 4 cm^–1^ using a Bruker Multipurpose Analyzer (MPA) at the World Agroforestry Centre (ICRAF) Plant and Soil Spectroscopy Laboratory in Nairobi, Kenya (http://worldagroforestry.org/research/land-health/spectraldiagnostics-laboratory).

Soil samples from all locations except DRC were analyzed for carbon concentration (% dry mass) using dry combustion on acidified samples and for stable carbon isotopes with an elemental analyzer isotope ratio mass spectrometer (EA-IRMS) at IsoAnalytics Laboratory (http://www.iso-analytical.co.uk). Soil samples from DRC were analyzed at Isotope Bioscience Laboratory (ISOFYS, www.ISOFYS.be) of Ghent University, Belgium using EA-IRMS (ANCA-SL (SerCon, Crew, UK), coupled to a 2020 IRMS (SerCon, Crew, UK)). Stable carbon isotopes were expressed as δ^13^C in parts per mile (‰ relative to the V-PDB (Pee Dee Belemnite) standard (Loomis and Connor 1992).

### NIR processing and prediction of SOC and δ^13^C

All calculations and statistical analysis were performed using R statistics (R Core Team 2015) and KNIME (Berthold et al. 2007). Manipulation of the NIR spectra included computing the first derivatives of the spectra using a Savitsky-Golay polynomial smoothing filter implemented in the *locpoly* function of the *KernSmooth* R package (Wand 2015). Partial Least Squares (PLS) regression analysis was conducted in R using the *pls* package and the *mvr* function (Mevik et al. 2015). A Random Forest (RF) model (Breiman 2001) was computed in R using the *randomForest* package (Liaw and Wiener 2002), while statistical analysis of between site differences was conducted using linear mixed effects models using the *nlme* package in R (Pinheiro et al. 2013).

Prediction models for δ^13^C were developed using PLS regression (Martens and Naes 1989), which is commonly used as a standard tool in chemometrics (Wold et al. 2001). In brief, PLS is a dimension reduction technique similar to classical canonical correlation analysis (CCA), but where covariance is maximized rather than correlation (Boulesteix and Strimmer 2007). The X- and Y-scores are chosen so that the relationship between successive pairs of scores is as strong as possible, similar to a robust form of redundancy analysis. Directions are sought in the factor space that are associated with high variation in the responses but biasing them toward directions that are accurately predicted (Tobias 1995). The PLS model was compared to a RF (Breiman 2001) prediction model. Random forests have a wide range of applications, both in classification and regression, and are increasingly used for multivariate calibration, including in soil science (Vågen et al. 2013a; Winowiecki et al. 2016a). An ensemble of 500 regression trees was built, where each tree was learned on a different set of observations in the input data and different combinations of NIR spectral wavebands.

## Results

### Description of sites

Basic site characteristics for the nine sites included in the study are shown in Table 1. The sites ranged in elevation from about 970 m for Didy in Madagacsar to about 1800 m for Ol Lentille, which is located in Laikipia County in central Kenya (Table 1). The sites ranged from wet tropical forests in Madagascar to dryland savanna in Kenya (Mpala, Ol Lentille and Kisping), but also included agriculturally dominated areas such as Burhale, Hoima, Luhihi, and Mbola. There were large variations in land use between the sites, with 69% of the sampled plots under cultivation in Burhale, compared to Mpala, Ol Lentille and Kipsing where there was no cultivation (Table 1). The latter represent shrubland/rangeland systems. In addition, we calculated the average tree densities for each site. Didy had the highest tree density (3910 trees ha^–1^) compared with Mega, which had the lowest (13 trees ha^–1^) (Table 1).

**Table 1:** Basic biophysical characteristics of the nine sites included in the study

Site	Average elevation (m)	% of the plots that were cultivated	Average tree density (tree ha^-1^)	Dominant Vegetation Classes
Burhale, DRC	1595	69	23	Cropland/Woodland
Didy, Madagascar	973	5	3910	Forest
Hoima, Uganda	1168	41	41	Cropland/Shrubland
Kipsing, Kenya	1219	0	111	Shrubland
Luhihi, DRC	1653	68	85	Cropland/Woodland
Mbola, Tanzania	1196	39	611	Cropland/Woodland
Mega, Ethiopia	1548	0.6	13	Shrubland/Grassland
Mpala, Kenya	1714	0	87	Shrubland/Grassland
Ol Lentille	1807	0	65	Shrubland

### NIR spectra, soil organic carbon (SOC) and carbon isotopic signatures

**Fig. 2:**
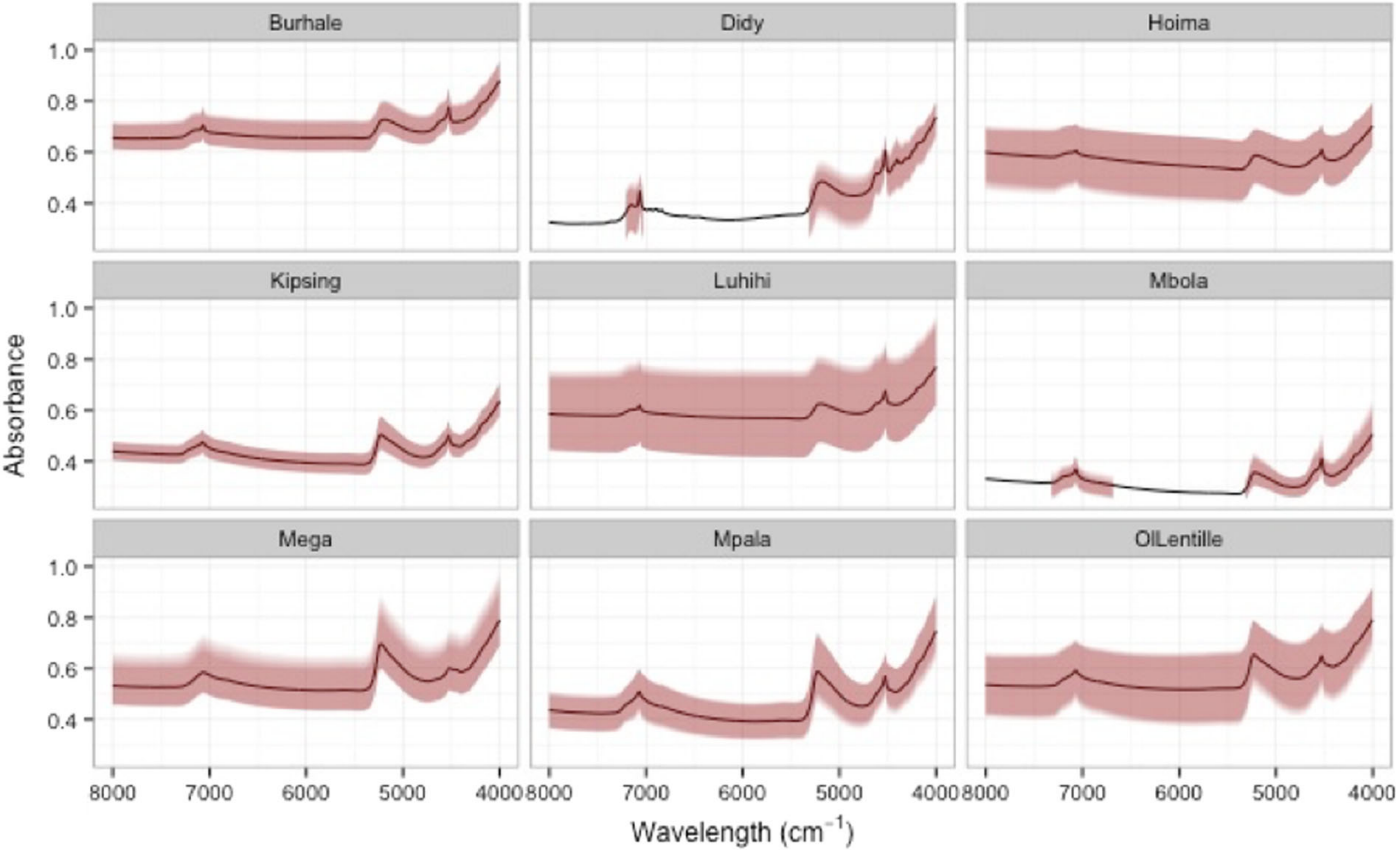
NIR spectra for the 156 topsoil samples from each of the nine LDSF sites. The black line is the mean spectra for the site and the shaded area is the standard deviation

There was substantial variation in soil NIR spectra both between and within the sites (Fig. 2). For example, Luhihi topsoil samples had high variation in absorbance within the site, especially compared to Didy, Mbola and Kipsing. The spectra of both top- and subsoil samples were used to develop calibration and validation models for δ^13^C in soil. Given the diversity of the spectra, the potential for applying these models across diverse landscapes is high.

**Fig. 3:**
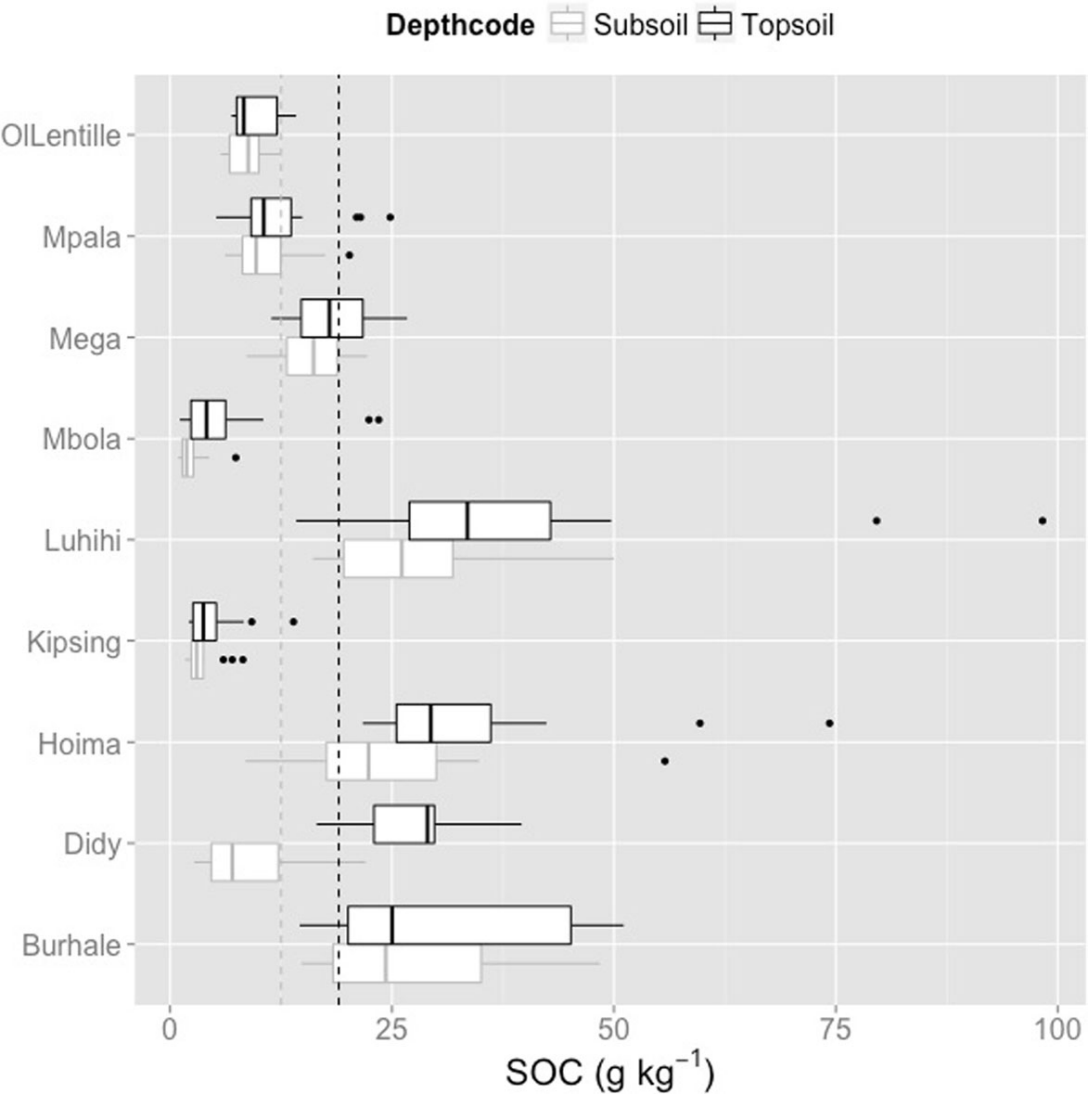
Boxplots of the top- and subsoil SOC variation for each of the nine LDSF sites. The black dotted vertical line is the mean topsoil OC across the sites, 18.9 g kg^–1^ and the gray dotted vertical line is the mean subsoil OC, 12.5 g kg^–1^

Soil organic carbon varied across the nine sites from 0.9 to 98.3 g kg^–1^. Mean topsoil OC across the sites was 18.9 g kg^–1^ and mean subsoil OC was 12.5 g kg^–1^. Figure 3 shows boxplots for topsoil and subsoil for each site (e.g., the median, 25th and 75th percentiles). Kipsing had the lowest median topsoil OC (3.8 g kg^–1^), followed by Mbola (4.1 g kg^–1^), OlLentille (8.3 g kg^–1^), Mpala (11.0 g kg^–1^), Mega (17.9 g kg^–1^), Burhale (25.0 g kg^–1^), Hoima and Didy (29.0 g kg^–1^), and then Luhihi (33.5 g kg^–1^) (Fig. 3). A comparison of the boxplots per site, not only indicate with sites had the highest SOC content, but also, which sites had the greatest variation within the site. For example, Didy had the greatest difference between top- and subsoil OC. Luhihi, Hoima, and Burhale had the highest variability in both top and subsoil SOC values within the site. Kipsing and Mbola had the smallest variation of SOC within the site, despite the variation in vegetation classes represented in Mbola.

**Fig. 4:**
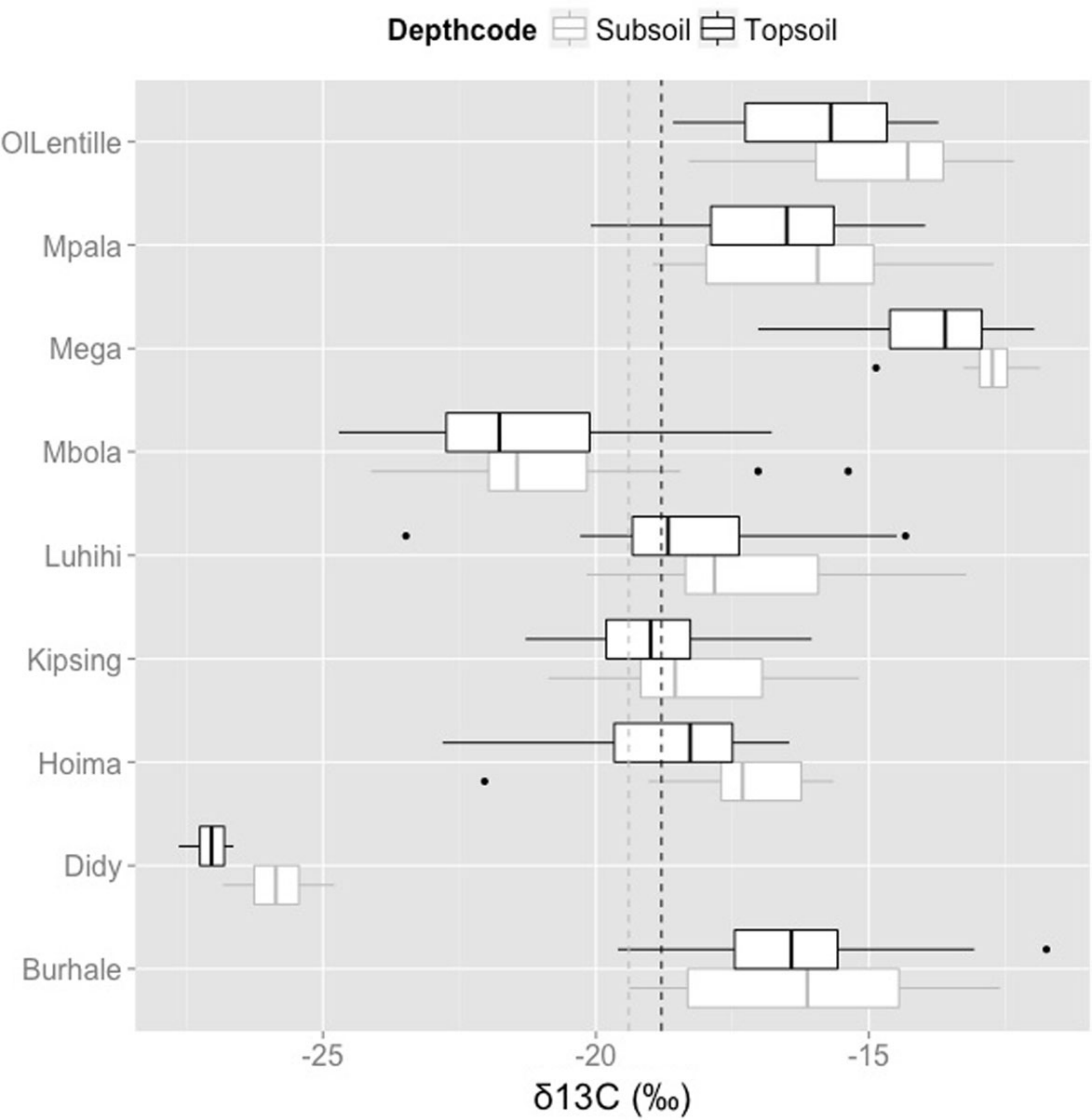
Boxplot of δ^13^C values for the top- and subsoil samples for each of the LDSF nine sites. The black dotted vertical line is the mean δ^13^C across the sites, –18.8‰ and the gray dotted vertical line is the mean subsoil δ^13^C, –19.4 ‰

Average δ^13^C in topsoil was –18.8‰, and average δ^13^C in subsoil was –19.4‰, which indicates that these are mixed C3-C4 ecosystems, with the exception of Didy, which is dominated by C3 vegetation (forest). Didy topsoil had the most negative δ^13^C values with a median of –27.04‰, followed by Mbola (–21.77‰), Kipsing (–19.00‰), Luhihi (–18.68‰), Hoima (18.27‰), Mpala (–16.50‰), Burhale (–16.42‰), OlLentille (–15.70‰), and Mega (–13.60‰) (Fig. 4). Furthermore, there was very little difference between top and sub soil δ^13^C values (with the exception of Didy), which may have implications for whether or not the organic matter is at steady state. However, the lack of a strong shift in isotopic signature with depth at most sites, could be due to the sampling intervals (0–20 and 20–50 cm), compared to sampling strategies that use smaller increments, or it could indicate that their have not been recent vegetative shifts.

### Vegetation structure class and δ^13^C values

**Fig. 5:**
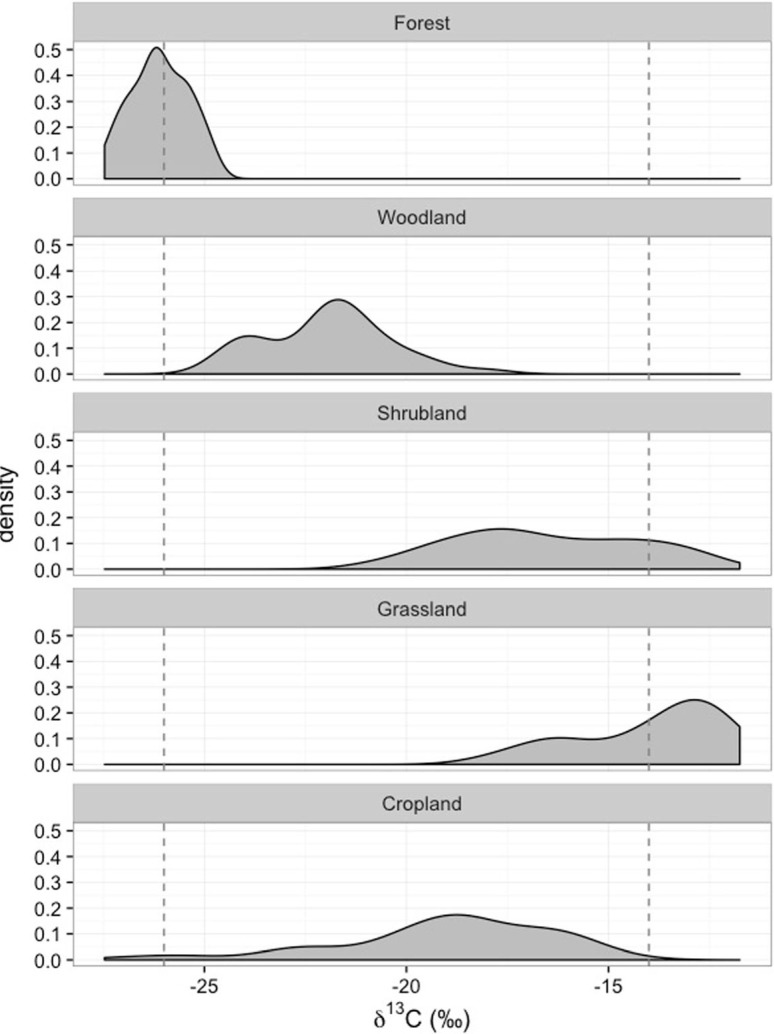
Density plots of δ^13^C values for the top- and subsoil samples for each of the five Land Cover Classifications (Forest (*n* = 56), Woodland (*n* = 41), Shrubland (*n* = 135), Grassland (*n* = 30), Cropland (*n* = 78). The black dotted vertical lines are average δ^13^C for C3 vegetation, –14‰ and for C4 vegetation, –26‰

Fifty-six plots were classified as forest, 41 plots as woodland, 135 plots as shrubland, 30 plots as grassland and 78 plots as cropland. Forested plots had the most negative average δ^13^C values (combining top and sub soil values), indicating a dominance of C3 vegetation, –26.1‰; followed by woodland, –21.9‰; cropland, –19.0‰; shrubland, –16.5‰; and finally grassland, –13.9‰ (Fig. 5). In general, the δ^13^C values for forested plots exhibited a C3 signature, while grassland plots exhibited a C4 signature. In contrast, the remaining vegetation classes exhibited a mixed C3-C4 signature. Results of the linear mixed effects models demonstrated that compared to the forest δ^13^C values, only shrubland (*p* < 0.05) and grassland (*p* < 0.001) plots showed significant difference. These data have important implications for the use of stable carbon isotopes in East Africa, e.g., since both semi-natural and cropland systems have mixed C3-C4 signatures large sample sizes will be needed to develop robust models to assess vegetation shifts and soil organic matter turnover rates.

### NIR prediction results for δ^13^C values

**Fig. 6:**
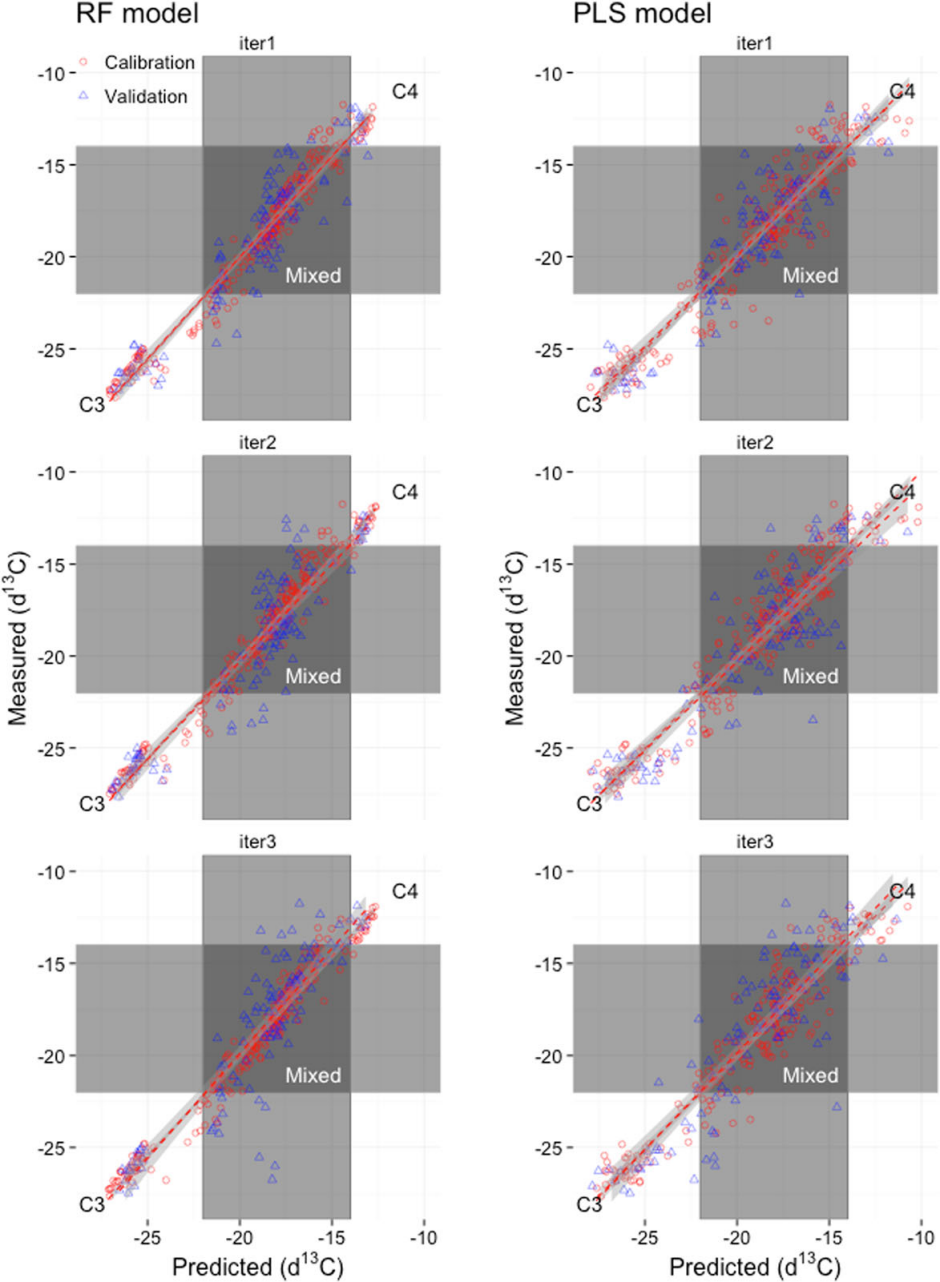
Measured vs. predicted δ^13^C values for the three calibration (red open circles) and validation (blue open triangles) runs using the Random Forest (RF) model (left panel) and the Partial Least Squares (PLS) regression model (right panel)

Prediction performance for δ^13^C was similar for the PLS and RF models when tested on three different validation datasets. Root Mean Square Error of Prediction (RMSEP), which is a useful measure of accuracy reflecting the overall difference between measured and predicted values, was low for both PLS and RF models (1.95 for PLS and 1.77 for RF). The results further show average R^2^ for the validation runs using PLS of 0.80 compared to a slightly higher average R^2^ using RF of 0.84. Overall, R values were slightly higher for calibration runs compared to validation runs, which is expected, with average R^2^ for calibration using PLS of 0.91 compared to 0.97 for RF. It is important to test prediction models such as the ones presented here on datasets that are independent of that used for developing the model in order to generate some measure of model stability. In our case, validation samples were drawn randomly in each iteration and the calibration model was fitted to this dataset with low RMSEP in both cases indicating good performance for both models (Table 2). Model stability was also good for both PLS and RF models (Fig. 6). Given the stability of the models, indicated by the similarity between the slopes of the regression lines in Fig. 6 and low RMSEP for the calibration and validation runs, respectively, there is significant potential for the application of RF models to large spectral libraries.

**Table 2:** Prediction performance for δ^13^C for three cross-validation (CV)runs for the calibration andvalidation datasets for the partialleast squares (PLS) and random forest (RF) models, expressed as the root mean squared error of prediction (RMSEP) and R^2^

CV run	Dataset	n	*RMSE*P_d^13^C_ RF	R^2^_d13C_ RF	*RMSE*P_d^13^C_ PLS	R^2^_d13C_ PLS
1	Calibration	226	0.92	0.97	1.33	0.90
	Validation	114	1.52	0.88	1.91	0.83
2	Calibration	227	0.86	0.97	1.24	0.91
	Validation	113	1.98	0.85	2.03	0.81
3	Calibration	227	0.87	0.97	1.34	0.91
	Validation	113	1.83	0.78	1.92	0.76

## Discussion

There is a need to better understand the global distribution of C3 and C4 plants for a number of different reasons, including for improving global circulation models for CO_2_, better assessing soil organic matter dynamics, as well as estimating water and energy cycles. Further, there is a need for better estimates of the spatial distribution of and temporal changes in plant communities with different photosynthetic pathways as plant communities respond differently to rising CO2 levels in the atmosphere, as well as to land degradation status. The nine sites used in this study had diverse vegetation classes and cropping systems, which is reflected in the wide range of SOC values (e.g., from 0.9 to 98.3 g kg^–1^). We found the highest SOC concentrations in Didy (eastern Madagascar), Burhale and Luhihi (eastern DRC) and Hoima (western Uganda), which all represent sub-humid and humid environments. The lowest SOC values were found in semi-arid environments (Ol Lentille, Mpala, Mega) and in Miombo woodlands (Mbola).

There are many factors influencing SOC concentrations, including inherent soil properties such as sand content, land degradation status such as erosion prevalence (Winowiecki et al. 2016a, 2016b), as well as land management practices (including burning in seminatural and cropland systems, fertilization, residue retention, among others) (Vanlauwe et al. 2015). The use of stable carbon isotopes can aid in better understanding SOC dynamics and the influence of vegetation shifts over time. Our results show that semi-natural woodland and shrubland systems, as well as cropland systems in East Africa had mixed C3-C4 signatures, while wet tropical forest plots in Madagascar exhibited a strong C3 signature and tropical grassland systems exhibited a C4 signature, as expected. Given that woodlands and shrublands contain both woody vegetation and grasses, such as in Miombo woodland systems with deciduous trees and an understory of tall perennial grasses, explains the mixed carbon isotopic signatures across most of the sites. Furthermore, woodland fragmentation is often driven by several competing activities including agricultural expansion, demand for forest resources, grazing, charcoal production, firewood collection and shifting cultivation practices (King and Campbell 1994; Sauer and Abdallah 2007; Syampungani et al. 2009). In croplands where farmers predominantly plant C4 species such as maize, we also saw a stronger C4 signature, depending on factors such as time since conversion in the case of areas that have been converted from natural forest. Examples of such sites in our study included Burhale in DRC and Hoima in Uganda. However, in cropland systems where tobacco, rice, soybean and maize are cultivated, the carbon signature is mixed, which increases the complexity in assessing the impacts on vegetation shifts using stable carbon isotopes.

In a chronosequence study from Madagascar, Vågen et al. (2006a) found a strong relationship between SOC and δ^13^C, with decreasing carbon going from C3-dominated systems (e.g., tropical forest) to C4- dominated vegetation (e.g., degraded grasslands and croplands) along a conversion gradient (Vågen et al. 2006a). However, in the current study we observe more mixed results and a low level of correlation between SOC and δ^13^C overall, partly due to the inclusion of more diverse (mixed) systems. In addition to conversion studies, Billings and Richter (2006) highlight the need for decadal studies in somewhat stable systems to better identify discrimination processes, again illustrating the complexity of stable isotopic pathways (Billings and Richter 2006).

Based on the validation predictions, model performances for predicting δ^13^C from NIR spectra were good both for RF (average RMSEP = 1.78) and PLS (average RMSEP = 1.95) models. Average R^2^ values for the three model validation runs were 0.84 and 0.80 for the RF and PLS models, respectively. Our results show that both PLS, which is a data-reduction technique, and RF, which is generally good for feature selection, can be successfully applied for the prediction of δ^13^C in soils. Further, the high performance of the RF model applied in our study indicates that this technique is suitable for detecting spectral features that are important for determining the relative abundance of ^12^C and ^13^C in soils. Viscarra Rossel and Behrens (2010) also evaluated a number of data mining techniques with NIR spectra, however they reported that PLS and support vector machines (SVM) outperformed RF, multivariate adaptive regression splines (MARS) and boosted regression trees (BT) (Viscarra Rossel and Behrens 2010). Exploratory application of remote sensing to map carbon isotopes was used in southern Africa (mixed C3-C4 systems), highlighting the need and potential solution to better understand ecosystem processes at larger spatial scales (Wang et al. 2010). Our results are similar to those reported by Fuentes et al. (2012), and given that we develop models across such a wide range of sites and spectral variation shows the potential of NIR spectroscopy for routine prediction of δ^13^C, including for the direct classification of soil samples into C3, Mixed or C4 carbon. Furthermore, we assessed the relationship between SOC and δ^13^C, and found that it was not linear. This confirms that the prediction accuracy of both the PLS and RF models are because we are able to predict δ^13^C (e.g., the weight of the isotope) and not merely predicting SOC. This further highlights the promise of spectroscopy for predicting stable isotopes in soil and shows the potential of new technological advances such as soil spectroscopic techniques for prediction of δ^13^C, which can lower the costs of analytical procedures and hence enable larger sample sizes and landscape-scale assessments of vegetation dynamics and SOC.

Building on these results, the potential use of mid-infrared spectroscopy (MIRS) should also be explored and is recommended by the authors for future studies. These results have important implications for the use of stable carbon isotopes in East Africa since both seminatural and cropland systems have mixed C3-C4 signatures. Hence, large sample sizes will be needed to develop robust models to assess vegetation shifts and soil organic matter turnover rates. In conclusion, application of spectroscopic techniques would allow for more costeffective analysis and increased sample sizes, which are needed for landscape-scale ecological studies.

## Abbreviations

δ^13^C: Delta carbon 13
BT: Boosted regression trees
ICRAF: World Agroforestry Centre
IRMS: isotope ratio mass spectrometry
LCCS: Land Cover Classification System
LDSF: Land Degradation Surveillance Framework
NIRS: Near infrared spectroscopy
MARS: Multivariate adaptive regression splines
MIRS: Mid-infrared spectroscopy
MPLS: Modified partial least squares
PLS: Partial least squares regression
RF: And random forest
RMSEP: Root mean square error of prediction
SOC: Soil organic carbon
SSA: Sub-Saharan Africa
UNCCD: The United Nations Convention to Combat Desertification
UNFCCC: United Nations Framework Convention on Climate Change
